# Identification of *CKX* gene family in *Morus* *indica* cv K2 and functional characterization of *MiCKX4* during abiotic stress

**DOI:** 10.1007/s44154-024-00173-x

**Published:** 2024-08-13

**Authors:** Chanchal Singhal, Arunima Singh, Arun Kumar Sharma, Paramjit Khurana

**Affiliations:** Department of Plant Molecular Biology, University of Delhi South Campus, Benito Juarez Road, New Delhi, 110021 India

**Keywords:** Cytokinin oxidases, Phylogeny, Gene duplication, Expression analysis, Drought stress

## Abstract

Cytokinin oxidase/dehydrogenase (CKX) is the key enzyme that has been observed to catalyze irreversible inactivation of cytokinins and thus modulate cytokinin levels in plants. *CKX* gene family is known to have few members which are, expanded in the genome mainly due to duplication events. A total of nine *MiCKXs* were identified in *Morus indica* cv K2 with almost similar gene structures and conserved motifs and domains. The *cis-*elements along with expression analysis of these *MiCKXs* revealed their contrasting and specific role in plant development across different developmental stages. The localization of these enzymes in ER and Golgi bodies signifies their functional specification and property of getting modified post-translationally to carry out their activities. The overexpression of *MiCKX4*, an ortholog of *AtCKX4*, displayed longer primary root and higher number of lateral roots. Under ABA stress also the transgenic lines showed higher number of lateral roots and tolerance against drought stress as compared to wild-type plants. In this study, the *CKX* gene family members were analyzed bioinformatically for their roles under abiotic stresses.

**Supplementary Information:**

The online version contains supplementary material available at 10.1007/s44154-024-00173-x.

## Introduction

Cytokinins are important plant hormones that contribute to the regulation of numerous biological processes such as cell division and differentiation, vascular and flower development, nutrient allocation, leaf expansion, root development, leaf senescence, and seed germination (Matsumoto-Kitano et al. 2008; Merewitz et al. 2010). Additionally, they help plants to militate against the severe consequences of abiotic and biotic stresses (Smigocki 1995; Liu et al. 2002; Babosha et al. 2009; Ikeda et al. 2021; Zhu et al. 2022; Argueso and Kieber 2024). Cytokinins (CKs) form a complex regulatory system along with other plant hormones such as auxin, gibberellins, abscisic acid, brassinosteroids, strigolactones and jasmonic acid (Hai et al. 2020). CKs are derivatives of purine bases with an isoprenoid or aromatic side chain at the N^6^ position. The natural isoprenoid CKs are *N*^*6*^-(Δ^2^-isopentenyl) adenine (iP), dihydrozeatin (DZ) and zeatin (*cis*-Zeatin and *trans*-Zeatin) whereas natural aromatic CKs include *ortho*-topolin, *meta*-topolin, 6-benzyladenine (6-BA) and 6-BA derivatives (Frébort et al. 2011; Argueso and Kieber 2024). The control of cytokinin levels in a particular tissue is important for its impact on growth and development. Homeostasis of CKs in cells is maintained via major three processes that are related to the rate of biosynthesis, inactivation/activation, and degradation. Isopentenyltransferases (IPT) are the major biosynthetic enzymes for the production of CKs via mevalonate (MVA) and methylerythritol (MEP) pathways. The inactivation and activation of CKs is a reversible process and primarily occurs through their conjugation with β-glucose, facilitated by enzymes such as zeatin O-glucosyltransferase (ZOG) and β-glucosidase (GLU), respectively. Meanwhile, the irreversible degradation of CKs and their derivatives is catalyzed by cytokinin oxidase/dehydrogenase (CKXs) encoded by a small multigene family in plants (Avalbaev et al. 2012; Jameson and Song 2016).

CKX is the only enzyme associated with CKs' breakdown to regulate their levels in plant development. CKX is a flavin adenine dinucleotide (FAD)–containing oxidoreductase that functions by oxidative cleavage of CKs carrying unsaturated *N*^*6*^-side chain, producing adenine (or its corresponding derivative for *N*^*9*^-substituted cytokinins) and a side chain-derived aldehyde (Sharma et al. 2022). Consequently, CKX regulates the level of free ribose of iP, zeatin, and also their ribosylated derivatives. Conversely, it is unable to act on CKs with saturated side chains like dihydrozeatin or *O-* glycosylated conjugates with glycosyl or xylosyl residues, and CKs with aromatic rings on their side chain. However, recent studies from *Arabidopsis thaliana* and *Zea mays* reported cleavage of aromatic cytokinins via CKXs although at a very low turnover rate when compared with that of iP (Galuszka et al. 2007; Frébortová et al. 2010). Earlier CKX activity was considered to be based on molecular oxygen, although many other electron acceptors were established to perform efficiently via other oxidizers and dehydrogenation. Thus, CKXs were classified as both cytokinin oxidase and cytokinin dehydrogenase (Galuszka et al. 2001). CKX enzymes comprise conserved FAD- and CK- binding domains located mainly on the N- and C-terminal of protein, respectively. The presence of a covalently bound FAD molecule is found in all known plant CKXs. The cofactor bonds through the 8-methyl group of the isoalloxazine ring to a histidine residue located in the conserved GHS motif in the N-terminal of the enzymes. CKX enzymes contain several consensus *N*-glycosylation sites that allow their post-translational modifications. This imparts CKXs regulation based on their enzymatic activity at different optimal pH i.e., 6.0 to 9.0, stability of protein, molecular weight, and localization. The presence of CKX inside or outside a cell thus depends highly on its specificity towards its substrate (Frébort et al. 2011).

Mulberry (*Morus* spp.) is a perennial, fast-growing, heterozygous, dioecious, and woody tree cultivated worldwide. *Morus* leaves are the major source of food with higher protein content for silkworm *Bombyx mori* which produces lustrous silk fibers. The use of mulberry for the production of silk makes it a significantly valuable tree for large-scale cultivation. Mulberry species like *Morus alba, Morus multicaulis,* and *Morus notabilis* are widely distributed in China. In India, *M. alba* is distributed in northern parts of the Himalayas and Punjab, while *M. indica* varies in range from temperate to sub-tropical and tropical regions. *Morus laevigata* is found naturally throughout India and *Morus serrata* is confined mainly to high-altitude regions and in northern India. The plantation of mulberry gets hampered by various unfavourable conditions including abiotic stresses such as heat, drought, and salt stress, as well as biotic stresses such as pests which destroy the plant completely. The draft genome sequence of *M. indica* cv K2 and RNA-seq data helped in the study of the expression of various genes in different developmental stages of plants (Jain et al. 2022a). In this particular study, we identified and studied *CKX* gene family members based on their structural, evolutionary, and functional analysis. *CKX* genes are reported to enhance tolerance against abiotic stresses including salt, drought, and heat in many plants. Therefore, we aimed to study the *CKX* gene family to provide a better understanding of mulberry growth and yield.

## Results

### Identification and sequence analysis of *CKX* in *M. indica* cv K2

The HMM approach was used for identifying the CKX proteins in *M. indica* cv K2 resulting in nine CKXs which were further confirmed using the SMART database and NCBI CDD. All nine MiCKXs were found to have two major conserved domains of CKXs, i.e., N-terminal FAD and C-terminal cytokinin binding domains (Fig. S1). The names of identified *MiCKX* were given according to the orthologs present in *A. thaliana*. The length of *MiCKX*s varied between 1506 to 1689 bp corresponding to their protein length ranging from 513 to 562 aa. The isoelectric points (pI) displayed variations from the lowest at 5.51 to the highest at 8.53. Other physiochemical properties like instability index, aliphatic index, and Grand average of hydropathicity (GRAVY) of MiCKX proteins were predicted. The subcellular localization predicted in different organelles and cytosol indicates their diverse role in cellular homeostasis. Additionally, the *N*-glycosylation sites of all MiCKXs were found to be present in the range of 2 to 4 (Table 1 and Table S1).

**Table 1:** Detailed information of *CKX* gene family in *M. indica* cv K2 with gene names and their respective IDs, scaffolds, gene start and stop position on respective scaffolds, protein length and molecular weights of proteins

Gene name	Gene ID	Scaffold	Gene start (bp)	Gene end (bp)	CDS length (bp)	Protein length (aa)	Mol. Wt. (kDa)
MiCKX5	Mi04721	Super-Scaffold_4_24552286	14,259,513	14,264,813	1650	549	61.07
MiCKX7.A	Mi05181	Super-Scaffold_4_24552286	20,461,212	20,465,965	1548	515	57.63
MiCKX7.B	Mi05287	Super-Scaffold_4_24552286	21,651,808	21,656,557	1548	515	57.617
MiCKX6.A	Mi11399	Super-Scaffold_9_20012512	7,839,637	7,843,485	1578	525	59.4
MiCKX6.B	Mi11459	Super-Scaffold_9_20012512	8,578,353	8,582,298	1579	553	62.63
MiCKX3	Mi18003	Super-Scaffold_15_15653510	12,279,811	12,282,808	1590	529	59.58
MiCKX4	Mi18004	Super-Scaffold_15_15653510	12,299,799	12,305,141	1506	513	57.83
MiCKX1.A	Mi22116	Super-Scaffold_24_6664442	2,732,468	2,734,977	1689	562	63.53
MiCKX1.B	Mi22130	Super-Scaffold_24_6664442	2,963,740	2,966,249	1689	562	63.53

### Gene structure, conserved and functional motifs

**Fig. 1:**
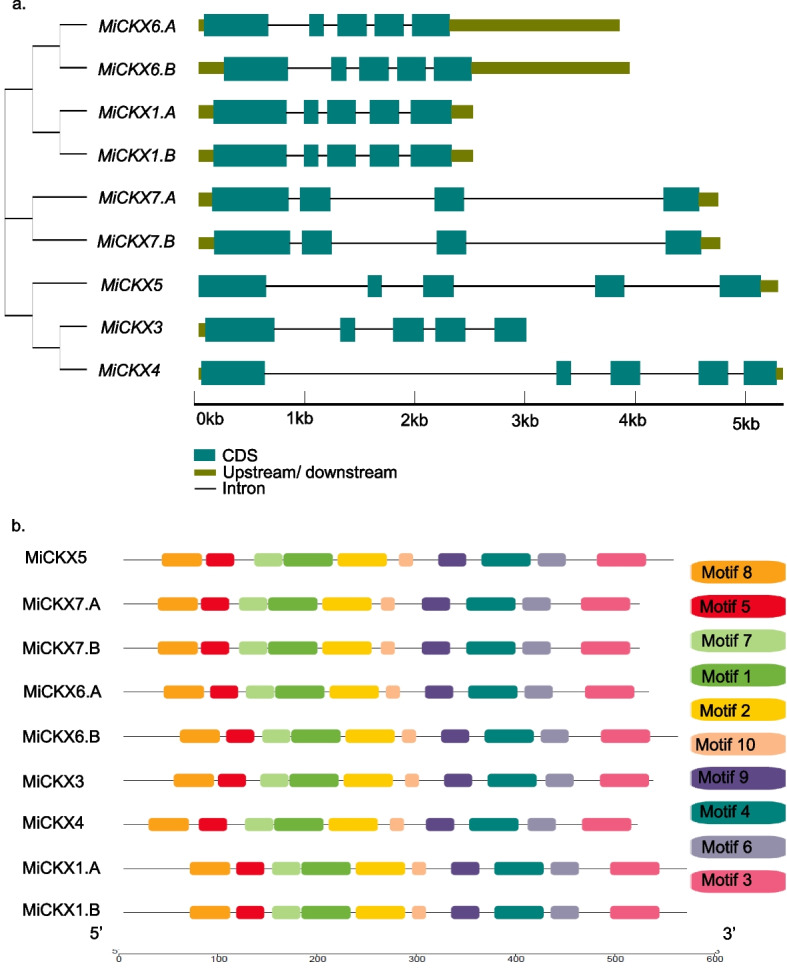
Gene structure and conserved motif analysis. **a** Illustration of the gene structure of nine *MiCKX* genes, showing the distribution of exons and introns. Exons are shown using solid boxes while introns are shown using solid lines. **b** Motif analysis

The gene structure deciphered not only the position of exons and introns but also helped in understanding the evolutionary factors that influence the function of CKX family members. The number of introns and exons were found to be 4 and 5, respectively in all *MiCKX*s except *MiCKX7.A* and *MiCKX7.B* where the introns were 3 and exons were 4 (Fig. 1a). The online software, MEME, predicted a total of 10 conserved motifs in all MiCKX proteins, designated as Motifs 1 to 10 (Fig. 1b).

### Multiple sequence alignment and phylogeny analysis

**Fig. 2:**
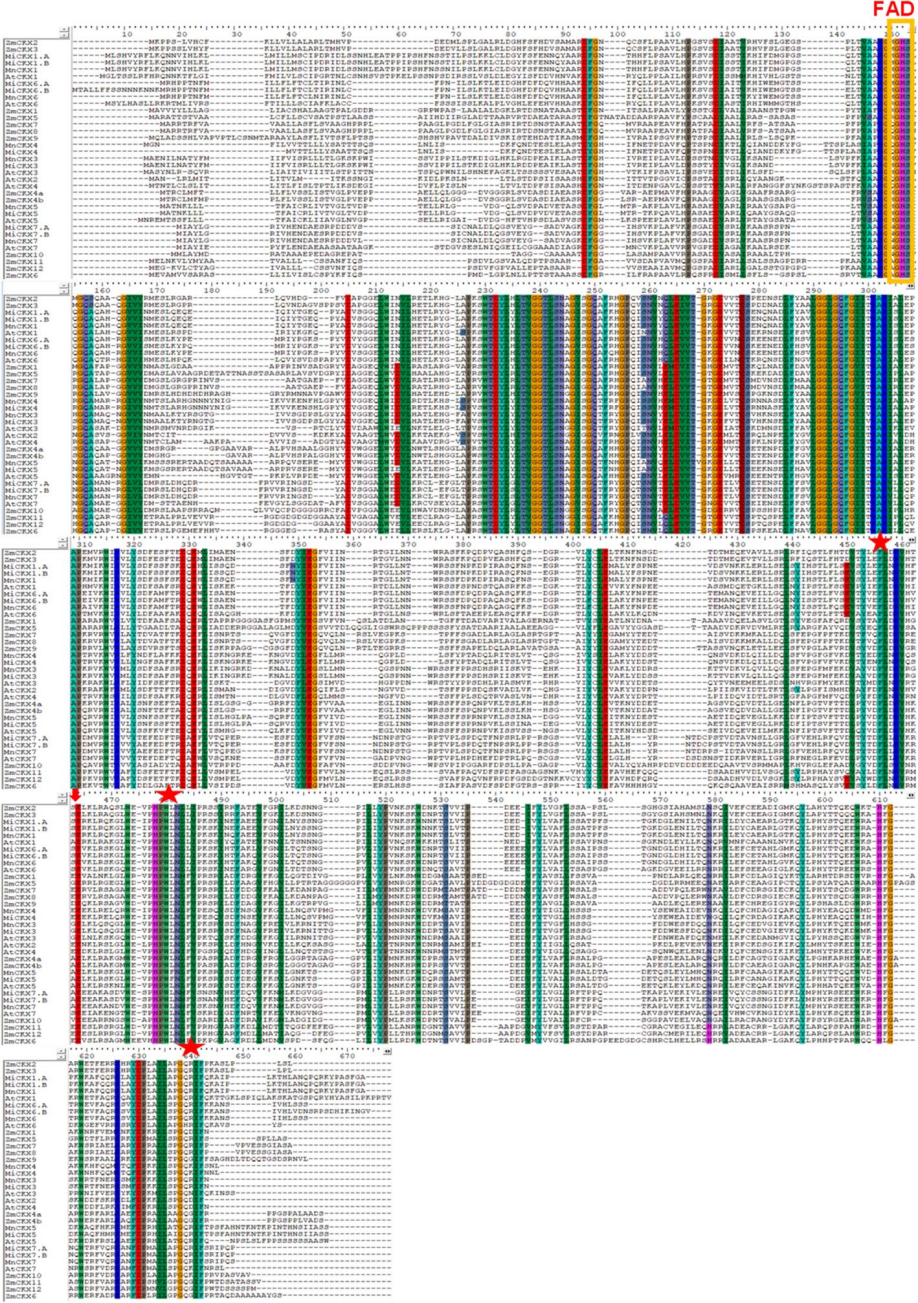
Alignment of amino acid sequences of MiCKXs. The position of the motif for covalent binding of FAD cofactor (GHS, where H is the binding residue in all plant CKXs) was indicated. Amino acid residues predicted to bind to the cytokinin substrate were marked with an asterisk. The cytokinin N9 hydrogen-bonding residue that affected the substrate specificity was marked with a red arrow

The sequence alignment of all MiCKX proteins along with ZmCKXs and AtCKXs proteins showed that FAD binding domain was well conserved among all known CKX sequences. Moreover, conserved motifs like GHS were also well conserved, where the FAD cofactor is linked through the 8-methyl group of isoalloxazine ring to the histidine residue (Frébort et al. 2011; Sharma et al. 2022). Further, motifs like PHPWLNL and PGQxIF were also found in all CKXs analyzed in this study. These conserved amino acid sequences suggest their potential functions in the recognition of substrate and electron transport (Fig. 2).

**Fig. 3:**
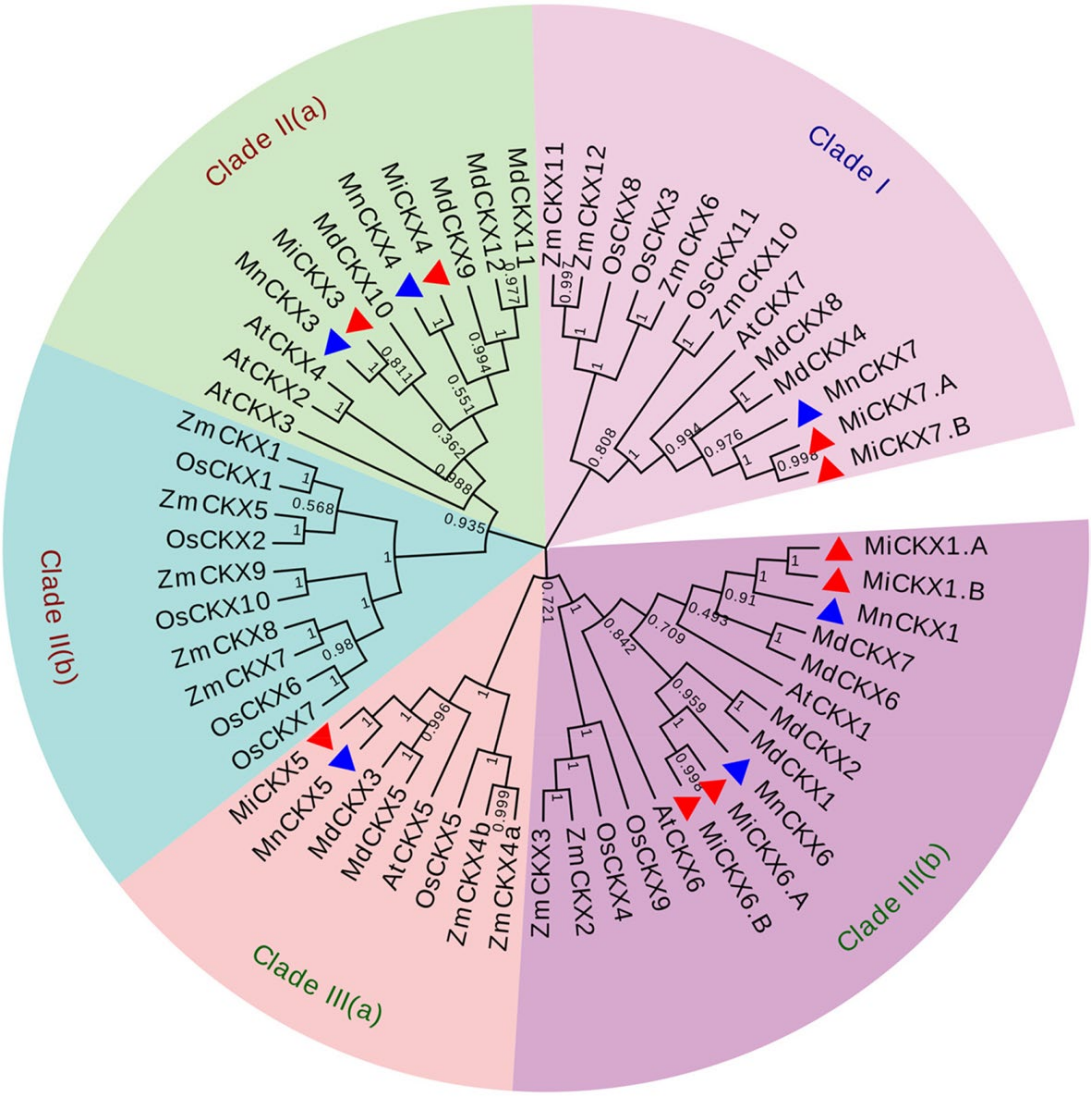
Phylogenetic analysis of CKX proteins. Amino acid sequences from *M*. *indica* cv K2, *M*. *notabilis*, *A. thaliana*, *Oryza sativa, Zea mays* and *Malus domestica* were aligned using MUSCLE and the phylogenetic tree was constructed using neighbor joining (NJ) method from MEGA-X. The numbers on the nodes denote bootstrap values from 1000 replicates. The tree here has been divided into four different clades.  indicates MiCKXs and  indicates MnCKXs

The aligned sequences were used for phylogenetic analysis and they were categorized into three main clades namely Clade I, II, and III. Clade I contained two MiCKXs i.e., *MiCKX7.A* and *MiCKX7.B* along with MnCKX7 and AtCKX7, 4 ZmCKXs, 2 OsCKXs, and 2 MdCKXs. The Clade II was divided into two subclades where Clade IIa contained CKX only from dicot species i.e., 3 AtCKXs, 4 MdCKXs, and 2 each from MiCKXs and MnCKXs; Clade IIb contained only monocot CKXs with 5 each of OsCKXs and ZmCKXs. Further, Clade III was also divided into two subclades which include Clade IIIa with 8 CKXs and Clade IIIb with 16 CKXs from all species (Fig. 3).

### Gene duplication and collinearity analysis

**Fig. 4:**
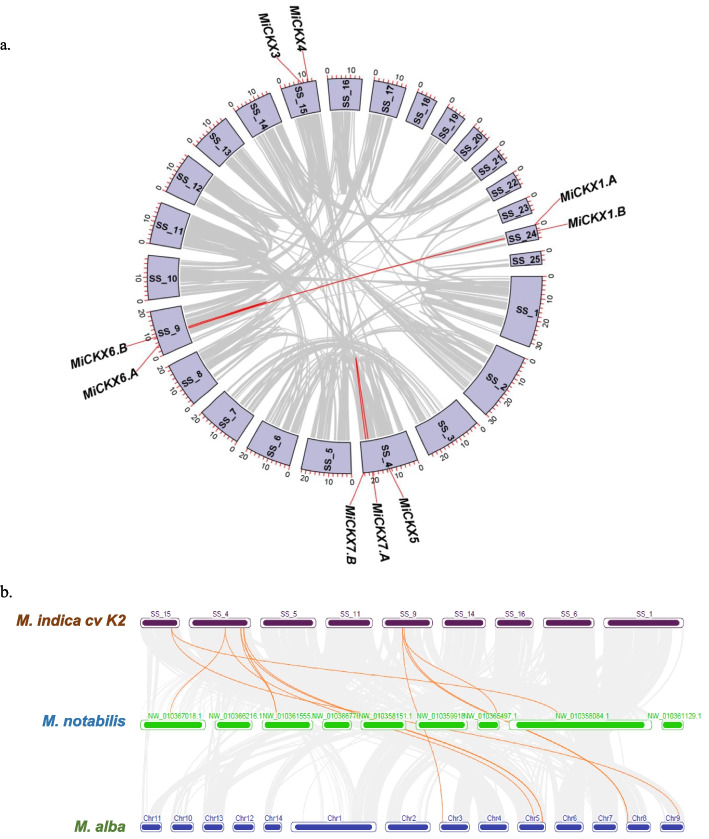
Duplication analysis of *MiCKX*s. **a** Mapping of duplicated *MiCKXs* on the Super Scaffolds (SS) and Scaffolds (S) using Circos. Grey ribbons represent collinear relationships among the blocks in the whole genome and red ribbons represent segmentally duplicated genes. **b** Synteny analysis of *CKX* genes *M*. *indica* cv K2 with *M. notabilis* and *M*. *alba*

The mechanism of gene duplication involves three major types of duplications i.e., proximal, tandem and segmental. In our study, three out of nine *MiCKX*s namely, *MiCKX7.A*/*MiCKX7.B*, *MiCKX6.A*/*MiCKX6.B* and *MiCKX1.A*/*MiCKX1.B* possessed highest similarity amongst each other. *MiCKX1.A*/*MiCKX1.B* are predicted to be a case of proximal duplication as the distance between the two is less than 20 genes and they are present on the same scaffold. *MiCKX7.A*/*MiCKX7.B* and *MiCKX6.A*/*MiCKX6.B* are predicted to be a part of tandem duplications as they are present on the same scaffold in more than 200 kb range (Fig. 4a). Collinearity analysis was performed between* Morus* genotypes i.e., *M. indica* cv K2, *M. alba* and *M. notabilis* for *CKX* genes to identify orthologs and characterize the evolutionary patterns of *MiCKX* genes within *Morus* genus. According to the synteny analysis, there are 7 orthologous *MiCKX* genes present in *M. alba* and 5 in *M. notabilis* (Fig. 4b).

### *Cis*-acting elements of *MiCKX*s promoter region

**Fig. 5:**
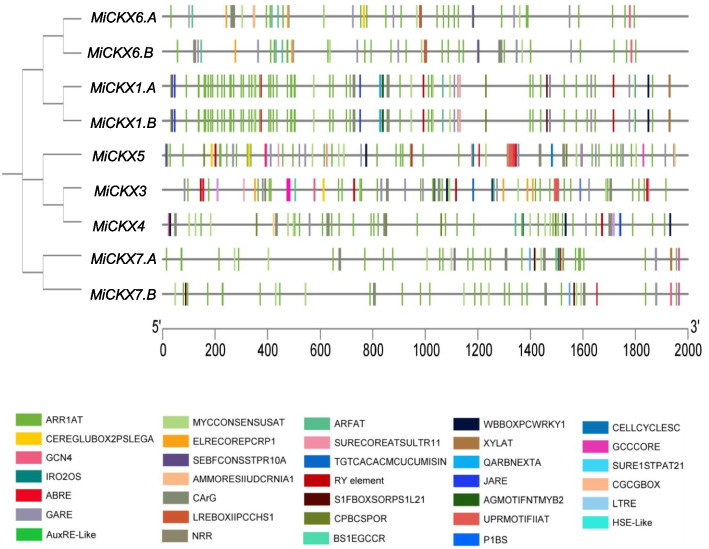
2 kb Promoter region of all nine MiCKXs in *M*. *indica* cv K2. Different colors represent different *cis*-elements

To reveal the potential role of *MiCKX* genes in various regulatory pathways of the plant life cycle, the promoter regions were studied. For this study, 2 kb upstream region of genes was taken, and based on the PLACE database, different elements were depicted that implicated the role of *MiCKX* in various stages of plant development. The promoter contained phytohormone-responsive responsive i.e., cytokinin and auxin-responsive elements, gibberellin, abscisic acid, and jasmonic acid-related elements. Cytokinin-mediated induction elements like ARR1 and CPBCSPOR were found which signifies their role in mediating cytokinin-related pathways. Various abiotic and biotic stress-related elements like drought, wounding, and defense implicated the role of *CKX* genes in the mitigation of different stresses. The identification of heat and low-temperature responsive elements along with light-related elements, further emphasizes the diverse regulatory functions of *MiCKX* genes. Additionally, the presence of distinct tissue-specific elements, such as those found in fruit, seed, and endosperm, denotes the fundamental significance of *CKX*s in tissue development processes in plants. Some of the distinct *cis-*elements were also observed. *MiCKX3*, *MiCKX1.A* and *MiCKX1.B* genes contained QARBNEXTA which is found in the promoter of *extA* extension genes and is induced under pathogenic attack (Hao et al. 2019). Moreover, there was one negative regulatory element NRRBNEXTA (Nakaminami et al. 2009) found only in *MiCKX3*. The element related to vascular development, XYLAT, was also found in *MiCKX7.A*, *MiCKX1.A* and *MiCKX1.B* along with BS1EGCCR in *MiCKX7.A* and *MiCKX7.B* indicating their role in vascular differentiation. The P1BS element related to phosphate starvation was found only in *MiCKX6.A* and IRO2OS element, induced exclusively in iron deficiency, was found in *MiCKX5*, *MiCKX7.A*, *MiCKX6.A* and *MiCKX6.B* implicating their role in nutrient deficiency conditions. One of the major *cis-*elements related to ER stress, UPRMOTIFIIAT, was also found in *MiCKX5* and *MiCKX3,* indicating their function in the unfolded protein response (UPR) which occurs in response to the accumulation of unfolded or misfolded proteins within the endoplasmic reticulum (ER) due to various changes in the cellular environment. The biotic stress-related elements like GCCCORE and WBBOXPCWRKY were also found, reinforcing the essential role of *MiCKX* genes in the plant defense mechanism against pathogen attack. The structural variation in the promoter regions of duplicated gene pairs, as in the case of *MiCKX7.A*/*MiCKX7.B* and *MiCKX6.A*/*MiCKX6.B*, highlights the differential regulatory pathways governing the roles of these genes in plant development (Fig. 5).

Gene ontology (GO) analysis gives detailed information regarding the three categories of gene properties, namely biological process, molecular function, and cellular components via GO terms. GO for all *MiCKXs* regarding the biological processes showed cytokinin and cellular hormone metabolic processes and regulation of hormone levels. The molecular functions enriched with GO terms suggest the role of CKXs in cytokinin dehydrogenase and oxidoreductase activity along with flavin adenine dinucleotide activity and ion binding activities. GO terms related to cellular components indicated their presence in the cytoplasm, intracellular part, and vacuoles. All these GO terms signify the role of *CKXs* in various aspects of plant growth and development (Fig. S2).

### Differential expression analysis of *MiCKXs* in different genotypes and tissues of *Morus*

**Fig. 6:**
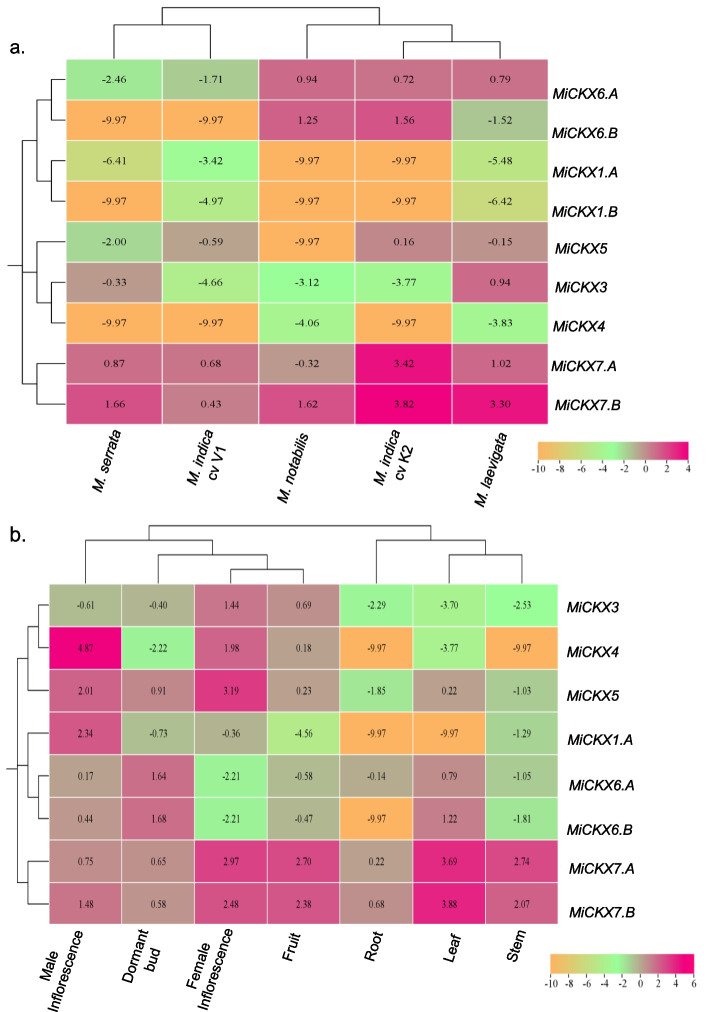
Heatmaps showing differential expression of *MiCKXs.* **a** Expression analysis in different genotypes of *Morus*. **b** Expression analysis in different developmental stages of *M. indica* cv K2

The expression patterns of *CKX* genes across different *Morus* genotypes and different tissues were analyzed using FPKM values procured from RNA-Seq data. In *M. indica* cv K2, amongst the nine *CKX* genes, four *MiCKX* genes, including *MiCKX1.A/MiCKX1.B*, *MiCKX3*, and *MiCKX4*, exhibited negligible expression levels. Conversely, *MiCKX7.B/MiCKX7.A* demonstrated the highest expression, followed by *MiCKX6.A*/*MiCKX6.B* and *MiCKX5*. These observations offer valuable insights into the differential expression patterns of *CKX* genes in *M. indica* cv K2, elucidating their variability in transcriptional regulation. Analogous expression profiles of *CKX* genes were observed in *M. laevigata,* with notable upregulation in *MiCKX7.A*/*MiCKX7.B*, followed by *MiCKX3* and *MiCKX6.A.* Noteworthy, *MiCKX3* displayed the highest expression level in *M. laevigata* compared to other genotypes, suggesting its specific involvement in the molecular landscape of this genotype. *MiCKX7.B*, followed by *MiCKX6.B*/*MiCKX6.A,* demonstrated the highest expression levels, among all other *MiCKX* genes in *M. notabilis*. Interestingly, the expression levels of *MiCKX7.A* were found to be lower in *M. notabilis*, contrasting with its higher expression in other genotypes, suggesting its distinct role in *M. notabilis.* In *M. serrata* and *M. indica* cv V1, all *MiCKX* genes displayed negligible expression levels, except *MiCKX7.B*/*MiCKX7.A,* which exhibited higher expression. Collectively, the differential expression of *MiCKX* genes highlights the functional diversification of these genes across *Morus* and indicates their possible role in local adaptation in response to various environmental conditions where these genotypes are flourishing (Fig. 6a).

The differential expression of genes across various tissues suggests their potential involvement in the development of different organs and implies their role in tailoring the needs and functions of different tissues within an organism. In this study, distinct patterns of *MiCKX* genes have been observed in *M. indica* cv K2, across various developmental stages which reflects their role in orchestrating specific processes at different stages of development during the plant growth. Amongst the nine *MiCKX* genes examined, *MiCKX* genes examined, *MiCKX7.B*/*MiCKX7.A* revealed a unique pattern of exclusive upregulation displaying the highest expression levels in the root and stem tissues, as compared to other *MiCKX* genes. This result emphasizes the possible importance of *MiCKX7.B*/*MiCKX7.A* in developing roots and shoots within the *Morus* genome. Additionally, in the leaf tissue, *MiCKX7.A*/*MiCKX7.B* showed elevated expression levels followed by *MiCKX6.B*/*MiCKX6.A* and *MiCKX5*, in contrast to the negligible expression levels observed for other *MiCKX* genes within the same tissue. Concerning dormant buds, *MiCKX6.A*/*MiCKX6.B* demonstrated the highest upregulation, followed by *MiCKX5* and *MiCKX7.A*/*MiCKX7.B*. Examining the female inflorescence tissues of *M. indica* cv K2, the expression levels of *MiCKX5* was the highest, followed by *MiCKX7.A*/*MiCKX7.B*. In the male inflorescence tissues, noteworthy elevation in the transcript levels was observed for all *MiCKX* genes except *MiCKX3*, with *MiCKX4* exhibiting the highest expression, followed by *MiCKX1.A* and *MiCKX5*. Moreover, it is important here to mention that *MiCKX1.A* showed higher expression specific to male inflorescence in contrast to other tissues, thereby, indicating its potential role in the development of male reproductive organs in *Morus*. In the fruit tissue, *MiCKX7.A*/*MiCKX7.B* displayed higher transcript levels as compared to *MiCKX3*, *MiCKX4,* and *MiCKX5*, whereas *MiCKX1.A* showed a negligible expression level (Fig. 6b). Altogether, the differential expression pattern of *MiCKX* genes provides insights into their role in respective tissues, warranting further investigation.

### Expression of *CKX* gene family at different hormonal levels and under drought stress

**Fig. 7:**
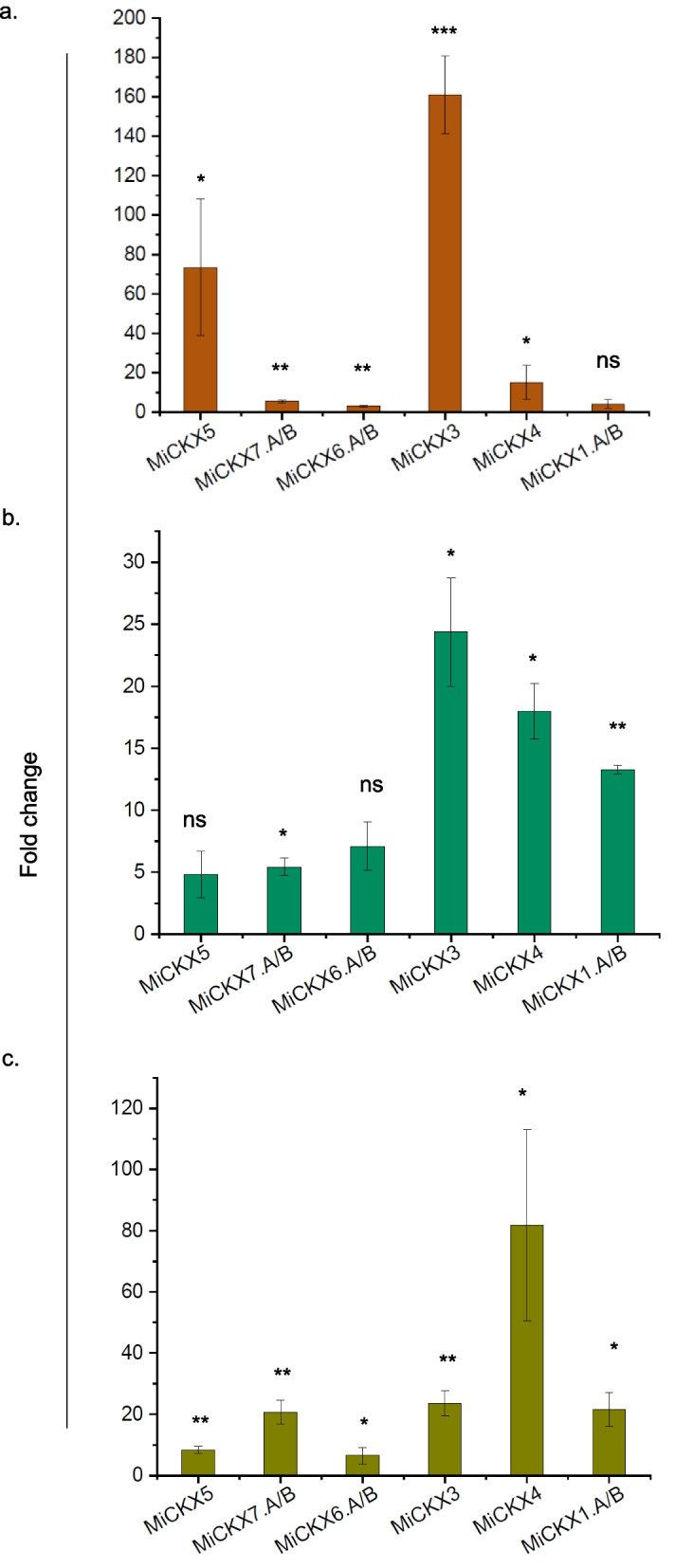
Relative expression analysis of *MiCKXs* under different conditions. **a** BAP (50 µM). **b** IAA (100 µM). **c** Drought stress via air drying of leaves. Three biological and three triplicates were used. Error bars indicate values ± SD. Asterisks on top of the error bars represent the significance levels (Student’s *t*-test; *p*-value ≤ 0.05); ns represents non-significant

The expression analysis at different hormonal and under drought stress for *MiCKX*s was performed using the RT-qPCR method that allowed monitoring of the expression of the target gene under different sets of conditions. The transcripts of three genes are *MiCKX7.A*/*MiCKX7.B*, *MiCKX6.A*/*MiCKX6.B,* and *MiCKX1.A*/*MiCKX1.B* are highly conserved among themselves and hence, these were analyzed jointly for both the copies. To study the effect of exogenous cytokinin, *MiCKX* transcript levels were checked after providing 6-Benzylaminopurine (BAP) at the concentration of 50 µM to leaves of* M*. *indica* cv K2 against the control conditions. The *MiCKX3*, followed by *MiCKX5* showed the highest upregulation under cytokinin treatment by 150 and 70 folds, respectively. Other *MiCKXs* were also found to be upregulated indicating the induction of *CKXs* upon higher levels of cytokinin in leaf tissue (Fig. 7a). In continuation with understanding the role of *CKXs* under hormonal stress, IAA treatment was given to the leaves at 100 µM concentration and it was observed that *MiCKX3* and *MiCKX4* showed maximum upregulation followed by *MiCKX1.A*/*MiCKX1.B* and *MiCKX7.A*/*MiCKX7.B* after the IAA treatment. The expression of *MiCKX6.A* and *MiCKX5* showed less upregulation as compared to other *MiCKXs* (Fig. 7b). The presence of ARF binding sites in the promoter region of *MiCKX3* and *MiCKX4* may contribute to this high expression. The results here suggest the crucial crosslink between the two hormonal signaling pathways.

To comprehend more about the purpose of CKX under abiotic stress and more specifically during drought stress, *Morus* leaves were air-dried and then analyzed for *CKX* transcript levels. *MiCKX4* was recorded to be highly upregulated, up to approximately 150-fold under drought stress evincing its indispensable role in conferring resistance against drought stress in *Morus*. *MiCKX3* was the second highly upregulated gene under drought stress followed by *MiCKX7.A/B* and *MiCKX.1/A* (Fig. 7c). The presence of elements which respond to drought stress probably relates to their higher expression levels.

### Subcellular localization of MiCKX proteins

**Fig. 8:**
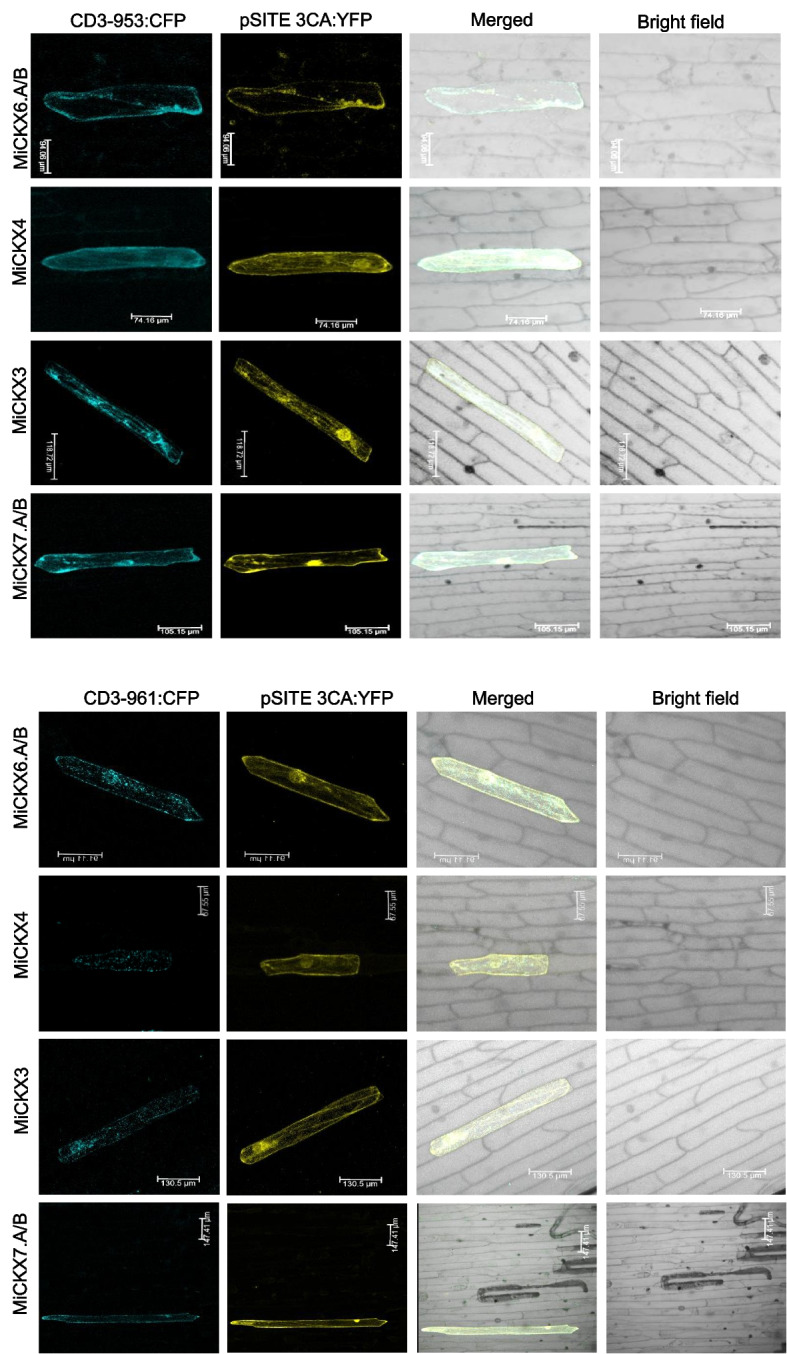
Subcellular localization of four MiCKXs with organelle markers. a Endoplasmic reticulum (CD3-953) marker. b Golgi bodies (CD3-961) marker. MiCKXs are tagged with YFP after cloning in pSITE3CA:YFP vector

The subcellular localization was analyzed for four MiCKXs via transient expression studies, using particle bombardment procedure in onion epidermal peels. The genes CDS were fused with YFP at the C-terminal. Expression of these chimeric genes was analyzed by and upon observation under confocal microscopy. The proteins were found to be present in cytoplasmic structures resembling endoplasmic reticulum (ER) and Golgi bodies. To validate these results, the co-expression of MiCKXs with ER and Golgi markers were checked. ER marker (ER-ck/CD3-953) and Golgi marker (G-ck/CD3-961) were used with CFP tags using appropriate gene constructs, respectively. The fluorescence was observed for both markers and gene samples, in both ER and Golgi bodies, confirming the localization of *MiCKX6.A/B*, *MiCKX3*, *MiCKX4*, and *MiCKX7.A/B* in these organelles (Fig. 8a and b).

### Overexpression of *MiCKX4* enhances drought tolerance

**Fig. 9:**
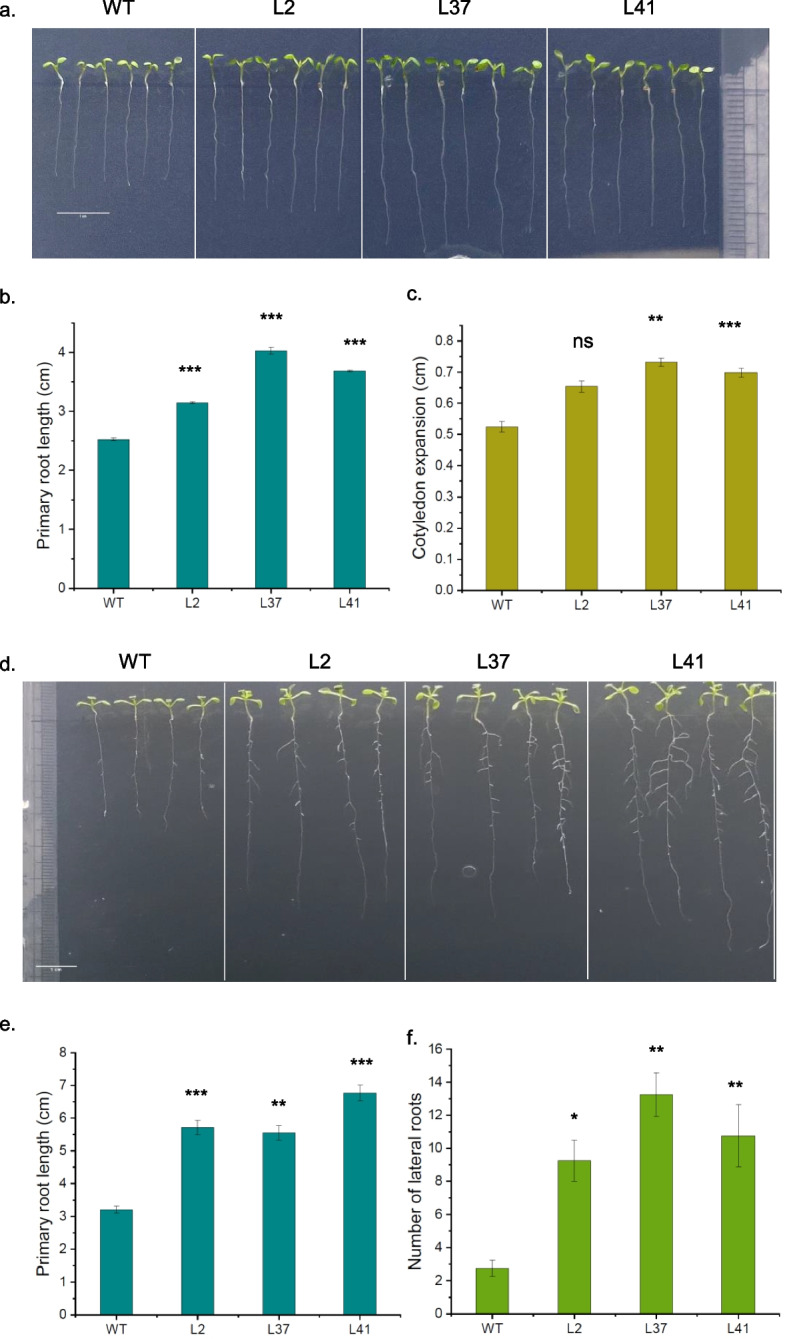
Phenotypic analysis of *MiCKX4* overexpression lines in *A. thaliana*. **a** Longer primary root length and larger cotyledon expansion in transgenics as compared to WT in 6-day-old seedlings. **b** and **c** Statistical analysis of primary root lenghth (**b**), cotyledon expansion (**c**). **d** Longer primary root length and higher number of lateral roots of transgenics as compared to WT in 10-day-old seedlings. **e** and **f** Statistical analysis of primary root length (**e**), number of lateral roots (**f**) **d**. Error bars indicate values ± SD. Asterisks on top of the error bars represent the significance levels (Student’s *t*-test; *p*-value ≤ 0.05); ns represents non-significant

**Fig. 10:**
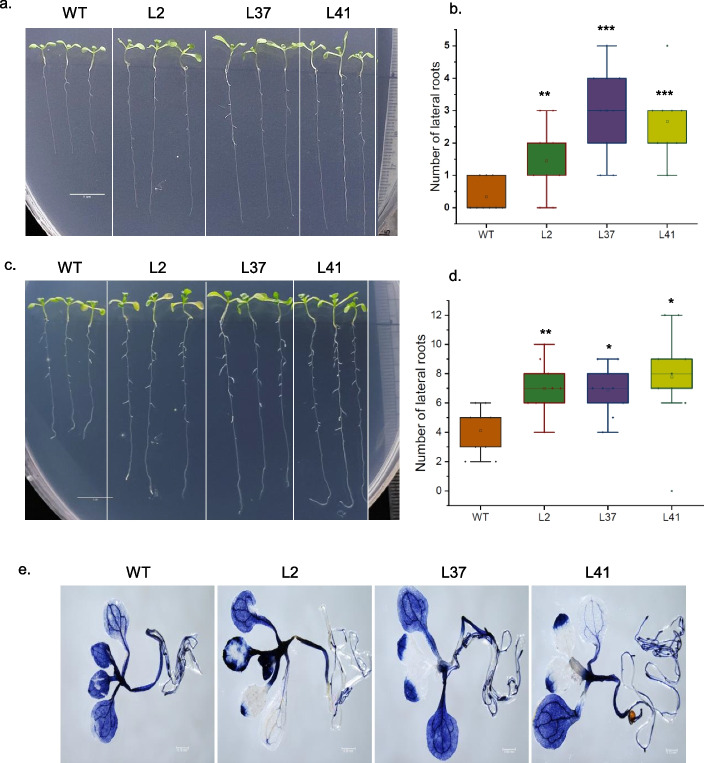
Phenotypic analysis of *MiCKX4* overexpression lines in *A. thaliana* in ABA (10 µM). **a** Higher number of lateral roots in transgenics as compared to WT in 6-day old seedlings before ABA treatment. **b** Statistical analysis of lateral root numbers of **a**. **c** Higher number of lateral roots in transgenics as compared to WT after 3 days of ABA treatment in transgenic plants. **d** Statistical analysis of lateral root numbers of **c**. **e** NBT assay for ABA-treated seedlings. Error bars indicate values ± SD. Asterisks on top of the error bars represent the significance levels (Student’s *t*-test; *p*-value ≤ 0.05); ns represents non-significant

To investigate the role of *MiCKX* genes in plants, transgenic Arabidopsis plants overexpressing *MiCKX4* were generated and three confirmed transgenic lines were used for analysis (Fig. S3). Under ½ MS media, the overexpression (OE) lines showed robust growth, characterized by longer primary roots and higher number of lateral roots as compared to wild-type (Fig. 9a and b). The expansion of cotyledon was also found greater in OE lines in comparison with wild-type Arabidopsis plants (Fig. 9c). The primary root phenotypes were noticed on the 6th day post-seed germination and analysis of lateral roots was carried out after the 10th day (Fig. 9d-f). To assess the drought stress tolerance of OE lines, the 6-day-old seedlings which were grown on ½ MS media, were transferred to ABA concentration of 10 µM and grown vertically for 2 days. The phenotypic analysis after two days of ABA stress revealed a notable increase in the number of lateral root production in OE lines as compared to wild-type plants under ABA-induced stress conditions. Additionally, the length of lateral roots was also significantly longer than the wild-type which indicates their role in conferring tolerance against drought stress mediated by ABA (Fig. 10a-d).

Reactive oxygen species (ROS) are the by-products of different metabolic pathways which are produced by the incomplete reduction of oxygen, thereby, producing highly reactive molecules. ROS act as important signaling molecules in response to stress and can be detected by NBT staining. NBT is an artificial electron acceptor and interacts with superoxide generated during various stresses. In our study, seedlings were subjected to ABA for 3 days followed by nitro blue tetrazolium (NBT) staining to assess the level of injury to the cell under stress conditions. The OE lines were found to have significantly lower amounts of ROS relative to wild-type under ABA stress (Fig. 10e). These results suggest that OE lines are producing less ROS and experiencing attenuated stress which contributes in enhancing drought tolerance in OE lines as compared to wild-type seedlings.

**Fig. 11:**
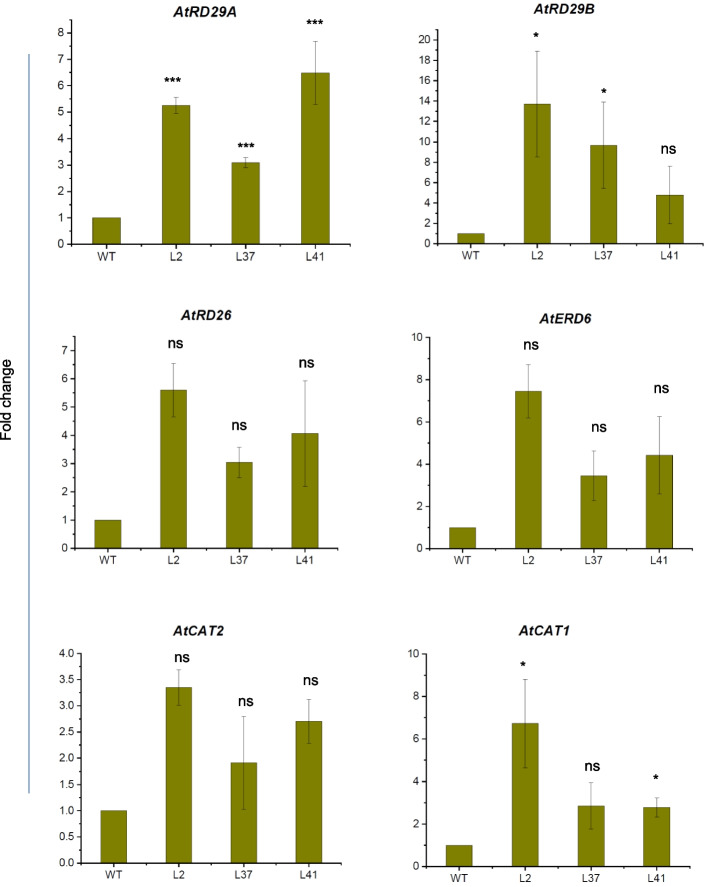
Relative expression analysis of drought stress marker genes. All the expression analyses were checked using RT-qPCR, where actin was used for normalizing values between WT and OE lines. Three biological and three triplicates were used for both control and treated samples. Error bars indicate values ± SD. Asterisks on top of the error bars represent the significance levels (Student’s *t*-test; *p*-value ≤ 0.05); ns represents non-significant

To illuminate more about the response of *MiCKX4* overexpression under drought stress, we checked the expression of stress-related genes that are known to get upregulated under multiple stress conditions in plants. The levels of *AtRD29A*, *AtRD29B*, *AtRD26,* and *AtERD6* were found to be higher in transgenics as compared to WT after ABA stress. Further, the levels of ROS-scavenging genes *AtCAT1* and *AtCAT2* were also higher in transgenics under stressed conditions (Fig. 11). These results suggest that *MiCKX4* imparts drought stress tolerance by the upregulation of stress-related genes and mediates ROS scavenging through the upregulation of antioxidant-generating enzymes.

## Discussion

Cytokinins are well known to display a significant impact at every stage of the plant life cycle by coordinating complex processes of growth involving differentiation, and morphogenesis by regulating development from roots to shoots, leaves to flowers, and fruits (Argueso and Kieber 2024). Moreover, extensive research has established the crucial role of cytokinins in plants under diverse environmental conditions including abiotic and biotic stresses (Argueso and Kieber 2024). *CKXs* are the major regulator of cytokinin in plants as they maintain the level of CKs via their irreversible degradation. Several members of the *CKX* gene family have been identified in various crops and their role in maintaining hormone homeostasis in the plants has been emphasized. In the present study, a total of nine *CKX* gene family members were identified which possess both FAD and cytokinins binding domain which are the signature domains of CKXs. The previous reports in *CKX* identification revealed that it is a gene family with a small number of members with conserved motifs and gene structure, which is also found in our study.

The exon–intron structure aids insights into the rate of gene expression and explores the diversification of functions associated with genes. The genes with the lower number of introns have been shown to rapidly change their expression levels in response to stress in Arabidopsis, yeasts, and mice (Jeffares et al. 2008). In the *MiCKX* gene family, the number of introns ranges from 3 to 4, which may contribute to their higher expression levels during various stress conditions. Moreover, the number of exons and introns was the same for all genes except, *MiCKX7.A/B*, suggesting the conservation of gene structure. The motif analysis helps in understanding the molecular and biological mechanisms of genes as these motifs serve as binding sites between molecules which affect the structure of molecules and provide proper enzymatic activities (Kemp 1980). In this study, MiCKX gene family members were found to have conserved motifs and domains that signify their functional conservation and evolutionary relationships amongst the gene family members.

The phylogenetic tree was constructed using amino acid sequences of five plant species (Arabidopsis, rice, maize, apple, and two genotypes of Morus) to explore their divergence during evolution. A previous study on *CKX* genes from *A. thaliana* reported seven AtCKXs, divided into three major clades (Schmülling et al. 2003). In this study, nine members of the *MiCKX* gene family were also divided into three major clades Clade I, II, and III with two sub-clades for Clade II and Clade III. The groups contained members with similar sequences and structures which conveys their similar role in plant development across the species. AtCKX7 is the only CKX present from Arabidopsis in Clade I which is considered one of the ancient CKX (Wang et al. 2020) in terms of its evolutionary analysis and the orthologs of AtCKX7 in other angiosperms are also considered ancient CKXs. Accordingly, we can predict MiCKX7.A and MiCKX7.B are the ancient CKX as they carry the same position in Clade I. All AtCKXs present in other clades were considered non-ancient. The Clade II contains two subclades that divide CKXs among monocots and dicots, indicating their genetic divergence during evolution and speciation. These non-ancient *CKXs* are known to provide tolerance to various abiotic stresses like drought, high temperature, salinity, etc. (Wang et al. 2020).

Gene duplication is an important mechanism that allows the expansion and diversification of gene families (Jaillon et al. 2009). The contribution of duplicated genes in producing genes with novel structures and functions, along with novel evolutionary traits has also been known for a role in crop improvement (Panchy et al. 2016). The genome sequence of *M*. *indica* cv K2 was revealed to consist of more than half of the genes duplicated through whole genome and tandem gene duplication events (Jain et al. 2022a). In this study, using circos analysis, however, we found that out of nine *MiCKX* genes, three are present as duplicated gene pairs due to proximal, tandem, and segmental duplication events. Further, the collinearity analysis between three genotypes of *Morus* revealed a closer relationship of *MiCKX* genes with *M*. *alba* followed by *M. notabilis.* The duplication events in *CKXs* suggest that functional redundancy may be more common among *CKX* genes in *M. indica* cv K2, which has also been found in other plant species like maize, rice, finger millet, etc., (Gu et al. 2010; Jain et al. 2022b; Blume et al. 2022).

Analysis of *cis*-regulatory elements renders understanding of the gene expression in deeper ways. In recent studies, promoters were considered to be the most important factor that may play a crucial role in crop improvement by regulating gene expression and influencing agronomic traits (Kummari et al. 2020; Villao-Uzho et al. 2023). The study of promoter sequences helps in understanding the role of a gene in various pathways by regulating their expression not only in a tissue-specific manner but also during abiotic and biotic stresses (Villao-Uzho et al. 2023). In this study, we utilized nucleotide sequences present 2000 bp upstream of the initiation codon of the gene and found various potential elements signifying the role of *CKXs* in various stages of plant development and stress mitigation pathways. Two major CK-induced elements i.e., ARR1AT and CPBCSPOR were found to be present in the promoters of *MiCKX* genes (Sakai et al. 2000; Ross et al. 2004; Fusada et al. 2005). The presence of more than 20 ARR1 sites in all *MiCKXs* makes it evident that they are induced highly by cytokinin and thus maintain cytokinin equilibrium in the plant. CPBCSPOR was found only in four *MiCKXs*, varying from one to three, further implicating their role in cytokinin-dependent events. Further, various other hormone-related elements like auxin, gibberellin, jasmonic acid, and abscisic acid were found in the promoter regions of *CKX,* indicating their roles in hormonal crosstalk. ARF binding sites present in *CKXs* promoter have been earlier reported to control crown root formation in rice. Moreover, the presence of various stress-related elements signifies the crucial role of *CKXs* in stress-mitigating pathways. Also, they contained elements related to iron and phosphate deficiency i.e., IRO2OS and P1BS (Rubio et al. 2001; Ogo et al. 2006), respectively, suggesting their roles in improving plant tolerance against nutrient deficiency, which is already reported in multiple crops like barley, rapeseed, and maize (Argueso and Kieber 2024). The tissue-specific elements further suggest the expression of *CKXs* is crucially regulated for the proper development of plant organs. It was also seen that duplicated genes except *MiCKX1.A*/*MiCKX1.B* do not share common elements suggesting functional divergences and the acquisition of novel biological functions of *MiCKX* genes.

Extensive studies related to the expression patterns of *CKXs* have been reported for maize and rice, where different *CKX*s have specific roles in maintaining cytokinin levels in different tissues (Schmülling et al. 2003; Rong et al. 2022). Expression analysis of *CKXs* in different developmental stages revealed their putative role in vegetative and reproductive development in *Morus*. Many *MiCKX*s exhibited distinguished tissue-specific expression patterns. *MiCKX7.A* and *MiCKX7.B* exhibited the highest expression in roots as compared to other *MiCKXs* which implies their role in root development. *AtCKX1* and *AtCKX2* were also found to play a major role in root development which was observed after their constitutive expression in *Nicotiana tabacum* (Khandal et al. 2020). Some *MiCKXs* were found to have higher expression in female and male inflorescence depicting the role of *CKXs* in flower development. Most of the *CKXs* had lower expression in dormant buds except *MiCKX4* and *MiCKX1.A* which revealed that cytokinin accumulation is a prerequisite for the growth of fruit. Further, the promoter activity exerts significant control over the regulation of gene expression, managing the complex process of turning genes on or off within a cell. The understanding of various regulatory pathways associated with a gene can be correlated with the presence of *cis*-elements within its promoter region. In our study, the expression levels of *MiCKX4* were observed to be very low in vegetative tissues including roots, stems, and leaves. This observation may be attributed to the presence of the unique *cis*-element NRRBNEXTA, a negative regulatory element known to modulate the development of roots, stems, and petioles, and alteration of this region leads to the expression of genes in tissues as mentioned above. Additionally, the presence of BS1EGCCR element, which is required for vascular expression and stem development, exclusively in *MiCKX7.A/MiCKX7.B*, demonstrates its elevated expression levels in the stem, distinguishing it from other *MiCKX* genes. Furthermore, hormone-related elements present in *MiCKX* genes associate their differential expression in vegetative and reproductive tissues which imply their role in the development and differentiation of various plant tissues.

The modulation of CKX enzyme activity seems to correlate with the diverse subcellular localization patterns demonstrated by these enzymes. Hence, the presence of intracellular CKX suggests its involvement in maintaining the movement of cytokinin within specific tissues or organs. Further, extracellular CKXs are anticipated to participate in degrading cytokinins that regulate the cell cycle (Avalbaev et al. 2012). In our study, four MiCKXs were studied for analysis of their subcellular localization, and their presence was detected in ER and Golgi bodies. The presence of MiCKXs in ER emphasizes their role in unfolded protein responses under stressed conditions, as detected earlier for AtCKX1 (Niemann et al. 2015, 2018). Further, their localization in Golgi bodies indicates their post-translational modifications as they are known to have N-glycosylation sites which contribute to regulation of their enzymatic activities, translocation, and protein stability (Frébort et al. 2011). Most of the CKXs in maize and other plants are glycoproteins with one to eight glycosylation sites (Schmülling et al. 2003). The pH difference was observed for glycosylated and non-glycosylated CKXs which suggests that the protein stability, enzymatic activity, localization, and translocation are highly dependent on the glycosylation properties of CKXs (Schmülling et al. 2003). However, in the present study, all nine MiCKXs were found to have glycosylation sites varying from a range of 2 to 4.

Genetic manipulation of *CKXs* has shown substantial improvement in agronomic traits by providing tolerance against various abiotic stresses that include salinity, drought as well as in nutrient deficiency. RNAi-mediated knockdown using *OsCKX2* delayed leaf senescence in transgenic lines along with the increase in panicle branches, grain number per panicle, and plant height, thereby, increasing seed yield (Yan et al. 2022). Further, CRISPR/Cas9 mediated gene knockout of *OsCKX11* also increased in tiller number, grains per panicle, and grains per plant while decreasing the fertility rate (Zhang et al. 2021). Further, the knockdown of *TaCKX1* and *HvCKX1* increased the number of spikes and overall grain yield in *Triticum aestivum* and *Hordeum vulgare*, respectively (Holubová et al. 2018; Jabłoński et al. 2020). In dicots like *A. thaliana*, *Brassica napus* and *Medicago truncatula*, mutants of *CKXs* produced a greater number of flowers and has larger flower sizes with a higher number of ovules per gynoecium, higher siliques and seed number per siliques (Bartrina et al. 2011; Schwarz et al. 2020; Wang et al. 2021). In terms of gain-of-function, the constitutive expression of *CKXs* in Arabidopsis*, Nicotiana, Medicago sativa*, etc., were observed to possess longer primary roots with increased number and length of lateral roots with dwarfed shoot phenotype and a smaller number of flowers (Sharma et al. 2022). The enhanced root growth indicates the importance of *CKXs* under drought and salinity stress as higher root biomass contributes to great tolerance against these stresses. Also, there are recent reports that indicate the vital role of *CKXs* in nutrient and minerals consumption in plants where the overexpression of the *CKX* gene resulted in higher content of various micro- and macro- elements which can be again a response due to increased size of the root systems (Sharma et al. 2022; Argueso and Kieber 2024). In the present report also, we see that overexpressed *MiCKX4* under the constitutive promoter in Arabidopsis resulted in robust root growth in transgenic lines as compared to wild-type Col-0. Both the primary roots and lateral roots exhibited increased length and number, respectively. Moreover, under simulated drought stress, transgenic lines displayed vigorous growth in lateral roots as compared to wild-type. The ROS production in transgenic lines was also very low as compared to WT, thus supporting the fact that overexpression of *MiCKX4* leads to lower stress in plants during drought conditions. Moreover, genes like *RD29A* and *Rd29B* (Msanne et al. 2011)*,* which are known to express very low under optimal conditions and get upregulated under stress conditions, were also found to express at higher levels in the transgenic lines as compared to wild-type. Our findings on *MiCKXs*, thus, provide more supporting evidence that *CKX* genes play an essential role in mitigating abiotic stresses and their function is conserved across diverse plant species.

## Conclusions

In the present study, we identified nine *MiCKXs* and found three duplicated genes which shows the expansion of the *CKX* gene family in *M. indica* cv K2. To get more understanding of the evolutionary significance, gene structure, phylogenetic relationship, and synteny as well as *cis*-elements were investigated for all *MiCKX* genes. The expression analysis of *MiCKXs* displayed variations in their levels during different developmental stages of plants thus suggesting their crucial role in plant organ development at both vegetative and reproductive stages. Additionally, the overexpression of the *MiCKX4* gene resulted in increased root mass, thereby conferring enhanced tolerance against drought stress.

## Materials and methods

### Identification and analysis of *MiCKX* genes

The information on the reference genome and annotated proteins of *M. indica* cv K2 was obtained from http://tgsbl.jnu.ac.in/MindGP/genome.php (Jain et al. 2022a) The Hidden Markov model (HMM) search (E-value equal to 10) and the Pfam database were used to screen the potential candidates for *MiCKX* proteins using FAD-binding domain and cytokinin binding domain with Pfam id PF01565 and PF09265, respectively. The retrieved sequences from HMM profiling were then taken forward to check the presence of conserved domains in these sequences. For this, two different software were used namely, NCBI-CDD and SMART, which analyze the presence of domains in the given protein sequences. The sequences with conserved FAD and cytokinin binding domains were then proceeded for further study. The protein sequences of the confirmed MiCKXs were then utilized to explore their physiochemical properties including molecular weight, isoelectric point (pI), instability index, aliphatic index, and GRAVY using the online available tool ProtParam (https://web.expasy.org/protparam/). Additionally, the *N*-glycosylation sites were determined using https://services.healthtech.dtu.dk/services/NetNGlyc-1.0/ for all MiCKX proteins.

### Gene structure analysis and conserved motif of MiCKX gene family

The CDS and genomic sequences of all *MiCKXs* were obtained and Gene Structure Display Server 2.0 (https://gsds.gao-lab.org/) was used to analyze the organization of exons and introns in *MiCKX* genes. The protein sequences of MiCKXs were utilized to understand the conserved motifs among CKXs using the MEME Suite 5.5.5 tool (https://meme-suite.org/meme/index.html) where the number of motifs was kept at 10 with optimum motif width between 8 and 50 residues. The derived result of motifs was further used in TBtools-II software to represent their conserved positions in MiCKX proteins (Chen et al. 2020).

### Phylogenetic analysis of the CKX Gene family

The multiple sequence alignment was performed using full-length protein sequences of various plant species consisting of monocots like *Zea mays*, *Oryza sativa,* and dicot species like *Arabidopsis thaliana*, *Malus domestica* and *Morus notabilis* against *Morus indica* cv K2. The protein sequences of 13 ZmCKXs, 11 OsCKXs, 7 AtCKXs, and 12 MdCKXs were retrieved using the Phytozome database (https://phytozome-next.jgi.doe.gov/) (Schmülling et al. 2003; Gu et al. 2010; Tan et al. 2018; Rong et al. 2022). The CKX protein sequences of *Morus notabilis* (MnCKXs) were searched by applying the HMM approach in the *Morus* Genome Database- MorusDB (https://morus.biodb.org/). Six MnCKXs with conserved FAD and cytokinin binding domains were found and used for analysis. ClustalW from MEGA-X was employed with default parameters for alignment and illustrated using BioEdit. The conserved FAD and cytokinin binding domains were highlighted in the alignment. The aligned file was used for constructing a phylogenetic tree using the neighbor-joining (NJ) tool of MEGA-X with bootstrap support of 1000 replicates. The Newick file output of the generated tree was uploaded to the online tool EvolView (https://www.evolgenius.info/evolview/#/treeview) for representation.

### Gene duplication and Synteny analysis of *MiCKX*

The duplication of *MiCKX* genes was studied using MCScanX tool available in Tbtools-II software (Wang et al. 2012). The file containing total linked genes present in *M*. *indica* cv K2 was generated and used to make CIRCOS using Advanced Circos application for selected super-scaffolds containing *MiCKX*s. The collinearity analysis was performed between three different *Morus* genotypes including *M. notabilis, M. alba,* and *M. indica* cv K2. The Multiple Synteny Plot modules of Tbtools-II were used to visualize the *CKX* gene among the above-mentioned species.

### Promoter and Gene Ontology analysis of *CKX* gene family

To identify the *cis-*elements in the promoter regions, the 2 kb region upstream from the start of the gene was retrieved for all *MiCKX* genes and used in the New PLACE database (https://www.dna.affrc.go.jp/PLACE/?action=newplace). Further, the Gene Ontology analysis was conducted using the PlantRegMap tool, and terms enriched for biological processes, molecular functions, and cellular components were studied. The expression levels of *CKXs* were checked using the transcriptome data present in http://tgsbl.jnu.ac.in/MindGP/genome.php for different genotypes and tissues of *Morus.* The FPKM values of *CKXs* were then used for representation using TB-ToolsII.

### Plant growth conditions and stress treatment

The freshly harvested seeds of *M. indica* cv K2 were imbibed overnight and then sterilized using 0.1% mercuric chloride for 8 min and subsequently washed with autoclaved RO water five times. The sterilized seeds were allowed to dry on Whatman paper for 2–3 h. The seeds were grown in MS media in a growth chamber maintained at 26 °C in the dark for 6 days. The germinated seeds were then shifted to 16/8 h light/dark period for further growth. The plants were allowed to grow till the four leaves stage and then transferred to pots. Two-month-old mature leaves were then used for various hormonal treatments and drought stress. For hormonal stress, IAA (100 µM) and BAP (50 µM) were used as auxin and cytokinin for 4 h, respectively. The leaves were air-dried for 4 h in the laminar hood to provide simulated drought stress. The tissues were then flash-frozen in liquid nitrogen and stored at -80 °C until further use.

### Subcellular localization of MiCKXs

The CDS of four *MiCKX*s were cloned in pENTR D-TOPO and the plasmid was sequenced. The clones were then transferred to the destination vector pSITE-3CA-YFP for detecting their subcellular localization. The individual plasmids DNA samples having the target gene or the organelle markers were coated with gold particles and bombarded at 900 psi pressure on the onion epidermal peels. The peels were observed under a confocal microscope (Leica, Germany) after 12 h. The organelle markers used for endoplasmic reticulum and Golgi bodies were ER-ck/CD3-953 and G-ck/CD3-961, respectively (Nelson et al. 2007).

### Generation of *MiCKX4* overexpression lines in *Arabidopsis thaliana*

The full-length coding region of *MiCKX4* was cloned in pENTR D-TOPO and confirmed via sequencing after which it was transferred to pMDC32 vector via Gateway cloning under CamV 35S promoter. The recombinant plasmid was transformed into *Agrobacterium* strain GV3101 and colonies were screened after 3 days. The wild-type Arabidopsis (Col-0) was transformed using the floral dipping method (Clough and Bent 1998). T_0_ transgenic seeds were screened on ½ MS containing 15 mg/L hygromycin for confirmation of plants with transgene. T_3_ homozygous seedlings were then confirmed for the presence of transgene by performing PCR of genomic DNA using *MiCKX4* specific primers and also via qPCR to check the transcript abundance of *MiCKX4* in transgenic lines. The confirmed lines were further utilized for analysis.

### Phenotypic study of *MiCKX4* overexpression lines under control and stress conditions

The wild-type seeds of Col-0 along with T_3_ seeds of *MiCKX4* overexpressing lines were sterilized using 0.4% sodium hypochlorite for 10 min and then washed with autoclaved RO five times. The sterilized seeds were then placed on ½ MS and kept on stratification at 4 °C in the dark for 2 days. On the third day, the seeds were transferred to the culture room at 22 °C with 16/8-h light/dark cycle. After 6 days of incubation, the primary root lengths were measured. The lateral root numbers were also calculated after 13 days of seed germination. For studying the stress tolerance, the 7-day-old seedlings of both Col-0 and *MiCKX4* overexpression (OE) lines were transferred to ABA concentrations of 10 µM. The phenotype was photographed after 3 days of stress.

### Histochemical analysis for ROS detection

The amount of reactive oxygen species (ROS) generated after ABA treatment in both wild-type Col-0 and *MiCKX4* OE lines was checked by staining the seedlings with nitro blue tetrazolium (NBT). The ABA-treated seedlings were incubated in NBT stain (NBT powder and 20 mM phosphate buffer) in the dark overnight. Next day the seedlings were transferred to a bleaching solution containing ethanol, acetic acid and glycerol in a ratio of 3:1:1 for chlorophyll removal. The seedlings were then visualized under the Leica S8 APO stereo microscope.

### RNA extraction for RT-qPCR analysis

The total RNA of mulberry leaves was extracted using RNeasy plant mini kit (Qiagen, Germany) according to the instructions provided by the manufacturer, including on-column DNaseI treatment for removing any genomic DNA contamination. Arabidopsis seedlings were isolated using Trizol reagent (Sigma), as per the manufacturer’s protocol. An aliquot having 2 µg RNA was then used for each sample to synthesize cDNA using the High-capacity cDNA Reverse Transcription kit (Applied Biosystems). The RT-qPCR analysis was performed using the ABI Prism 7000 Sequence Detection System and Software on ABI QuantStudio™ 3 Real-Time PCR machine. The obtained Ct values were used to calculate relative expression levels via the 2-ΔΔCt method, using Actin as internal control gene. The primers used are listed in Table S2.

### Statistical analysis

The GraphPad Prism 8 was used to perform a *t*-test for evaluating the statistical significance. *P *< 0.05, 0.01, and 0.001 were regarded as significant, very significant, and extremely significant, respectively, from the control conditions.

### Supplementary Information

Additional file 1: Table S1. Information on MiCKX proteins with their physiochemical parameters with pI, instability index, aliphatic index, and GRAVY along with their subcellular localization and N-glycosylation.

Additional file 2: Fig. S1. Domain analysis of MiCKXs depicting FAD and cytokinin binding domains. Fig. S2. Gene ontology analysis. a Biological processes. b Molecular processes. c Cellular components. Fig. S3. Transgenic confirmation of MiCKX4 overexpression lines in *A*. *thaliana* Col-0 Wild type. a Genomic DNA confirmation using MiCKX4 gene-specific primers. b Relative transcript levels of MiCKX4 in transgenics as compared to wild-type.

## Data Availability

Not applicable.

## Abbreviations

ABA: Abscisic acid
ARR: Arabidopsis Response Regulator
ARF: Auxin Response Factor
BAP: 6-Benzylaminopurine
CAT1: Catalase 1
CAT2: Catalase 2
CDS: Coding sequences
CFP: Cyan Fluorescent Protein
CK: Cytokinins
CKX: Cytokinin oxidases/dehydrogenases
ER: Endoplasmic reticulum
ERD6: Early response to dehydration 6
FAD: Flavin adenine dinucleotide
FPKM: Fragments Per Kilobase
GRAVY: Grand average of hydropathy
GO: Gene ontology
HMM: Hidden Markov model
IAA: Indole-3-acetic acid
MEME: Multiple Em for Motif Elicitation
NCBI-CDD: National Center for Biotechnology Information Conserved Domain Database
OE: Overexpression
PLACE: Plant cis-acting regulatory DNA elements
RD29A: Responsive to desiccation 29A
RD29B: Responsive to desiccation 29B
ROS: Reactive oxygen species
RT-qPCR: Reverse transcription-quantitative polymerase chain reaction
SMART: Simple Modular Architecture Research Tool
YFP: Yellow Fluorescent Protein
