# Supplementary material for: Identification of CKX gene family in Morus indica cv K2 and functional characterization of MiCKX4 during abiotic stress
Source: Stress Biol. 2024 Aug 13;4(1):35. doi: 10.1007/s44154-024-00173-x (PMC11322459; doi:10.1007/s44154-024-00173-x)
Supplement: Supplementary file 1 — Additional file 1: Table S1. Information on MiCKX proteins with their physiochemical parameters with pI, instability index, aliphatic index, and GRAVY along with their subcellular localization and N-glycosylation. [file 44154_2024_173_MOESM1_ESM.pptx]

## Slide 1
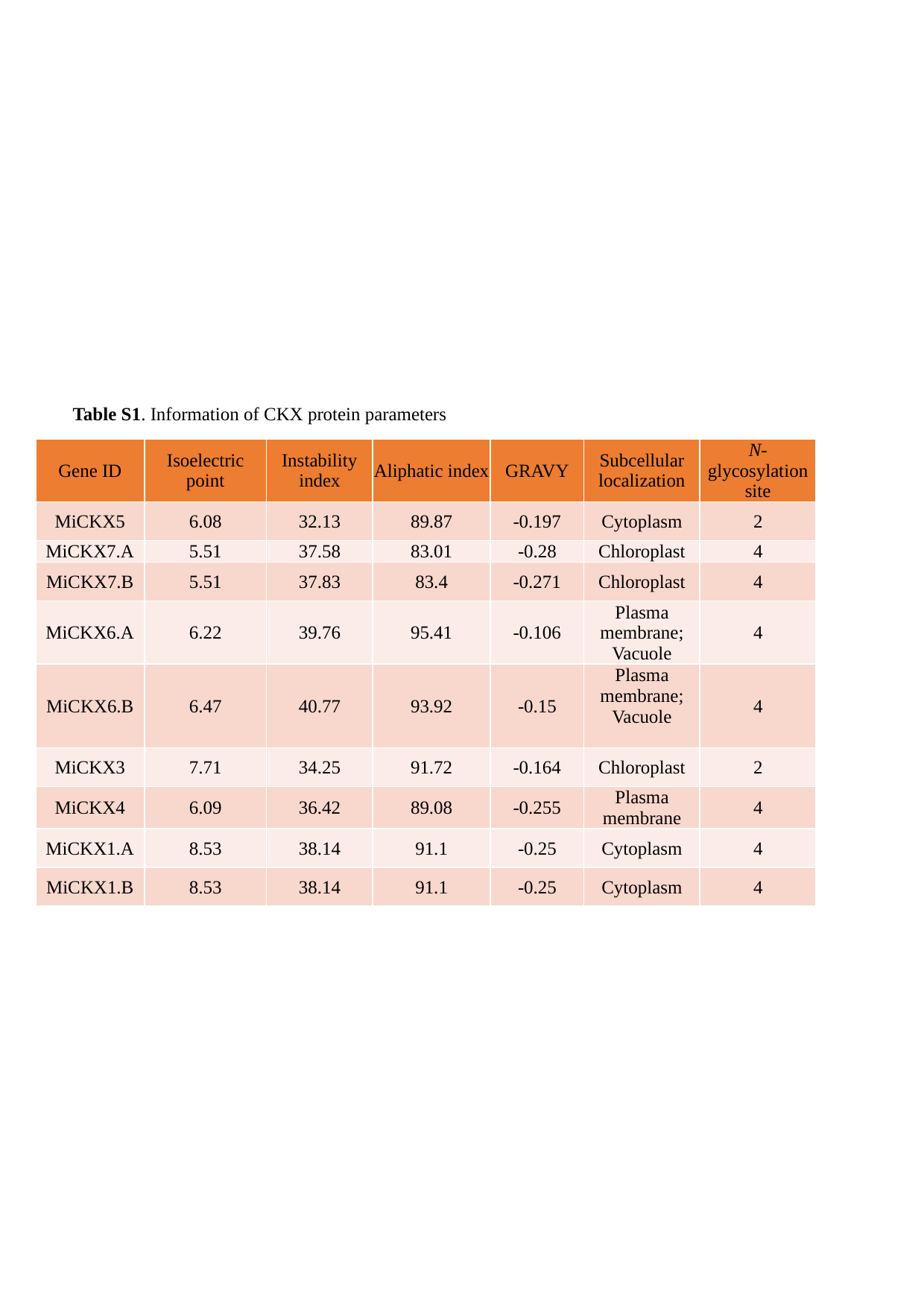

Table S1. Information of CKX protein parameters
| Gene ID | Isoelectric point | Instability index | Aliphatic index | GRAVY | Subcellular localization | N-glycosylation site |
| --- | --- | --- | --- | --- | --- | --- |
| MiCKX5 | 6.08 | 32.13 | 89.87 | -0.197 | Cytoplasm | 2 |
| MiCKX7.A | 5.51 | 37.58 | 83.01 | -0.28 | Chloroplast | 4 |
| MiCKX7.B | 5.51 | 37.83 | 83.4 | -0.271 | Chloroplast | 4 |
| MiCKX6.A | 6.22 | 39.76 | 95.41 | -0.106 | Plasma membrane; Vacuole | 4 |
| MiCKX6.B | 6.47 | 40.77 | 93.92 | -0.15 | Plasma membrane; Vacuole | 4 |
| MiCKX3 | 7.71 | 34.25 | 91.72 | -0.164 | Chloroplast | 2 |
| MiCKX4 | 6.09 | 36.42 | 89.08 | -0.255 | Plasma membrane | 4 |
| MiCKX1.A | 8.53 | 38.14 | 91.1 | -0.25 | Cytoplasm | 4 |
| MiCKX1.B | 8.53 | 38.14 | 91.1 | -0.25 | Cytoplasm | 4 |

## Slide 2
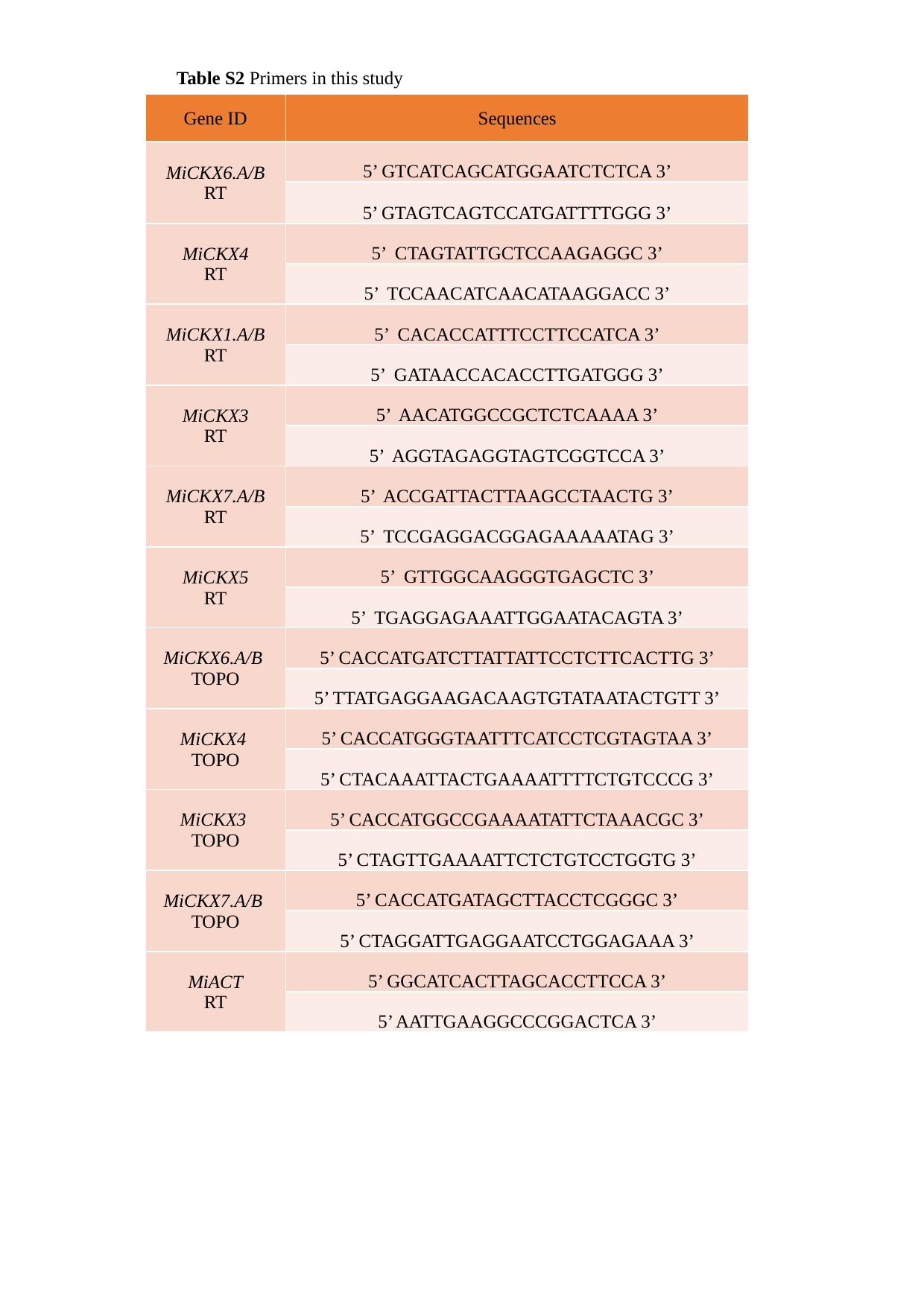

Table S2 Primers in this study
| Gene ID | Sequences |
| --- | --- |
| MiCKX6.A/B RT | 5’ GTCATCAGCATGGAATCTCTCA 3’ |
| | 5’ GTAGTCAGTCCATGATTTTGGG 3’ |
| MiCKX4 RT | 5’ CTAGTATTGCTCCAAGAGGC 3’ |
| | 5’ TCCAACATCAACATAAGGACC 3’ |
| MiCKX1.A/B RT | 5’ CACACCATTTCCTTCCATCA 3’ |
| | 5’ GATAACCACACCTTGATGGG 3’ |
| MiCKX3 RT | 5’ AACATGGCCGCTCTCAAAA 3’ |
| | 5’ AGGTAGAGGTAGTCGGTCCA 3’ |
| MiCKX7.A/B RT | 5’ ACCGATTACTTAAGCCTAACTG 3’ |
| | 5’ TCCGAGGACGGAGAAAAATAG 3’ |
| MiCKX5 RT | 5’ GTTGGCAAGGGTGAGCTC 3’ |
| | 5’ TGAGGAGAAATTGGAATACAGTA 3’ |
| MiCKX6.A/B TOPO | 5’ CACCATGATCTTATTATTCCTCTTCACTTG 3’ |
| | 5’ TTATGAGGAAGACAAGTGTATAATACTGTT 3’ |
| MiCKX4 TOPO | 5’ CACCATGGGTAATTTCATCCTCGTAGTAA 3’ |
| | 5’ CTACAAATTACTGAAAATTTTCTGTCCCG 3’ |
| MiCKX3 TOPO | 5’ CACCATGGCCGAAAATATTCTAAACGC 3’ |
| | 5’ CTAGTTGAAAATTCTCTGTCCTGGTG 3’ |
| MiCKX7.A/B TOPO | 5’ CACCATGATAGCTTACCTCGGGC 3’ |
| | 5’ CTAGGATTGAGGAATCCTGGAGAAA 3’ |
| MiACT RT | 5’ GGCATCACTTAGCACCTTCCA 3’ |
| | 5’ AATTGAAGGCCCGGACTCA 3’ |
