# Supplementary material for: Identification of CKX gene family in Morus indica cv K2 and functional characterization of MiCKX4 during abiotic stress
Source: Stress Biol. 2024 Aug 13;4(1):35. doi: 10.1007/s44154-024-00173-x (PMC11322459; doi:10.1007/s44154-024-00173-x)
Supplement: Supplementary file 2 — Additional file 2: Fig. S1. Domain analysis of MiCKXs depicting FAD and cytokinin binding domains. Fig. S2. Gene ontology analysis. a Biological processes. b Molecular processes. c Cellular components. Fig. S3. Transgenic confirmation of MiCKX4 overexpression lines in A. thaliana Col-0 Wild type. a Genomic DNA confirmation using MiCKX4 gene-specific primers. b Relative transcript levels of MiCKX4 in transgenics as compared to wild-type. [file 44154_2024_173_MOESM2_ESM.pptx]

## Slide 1
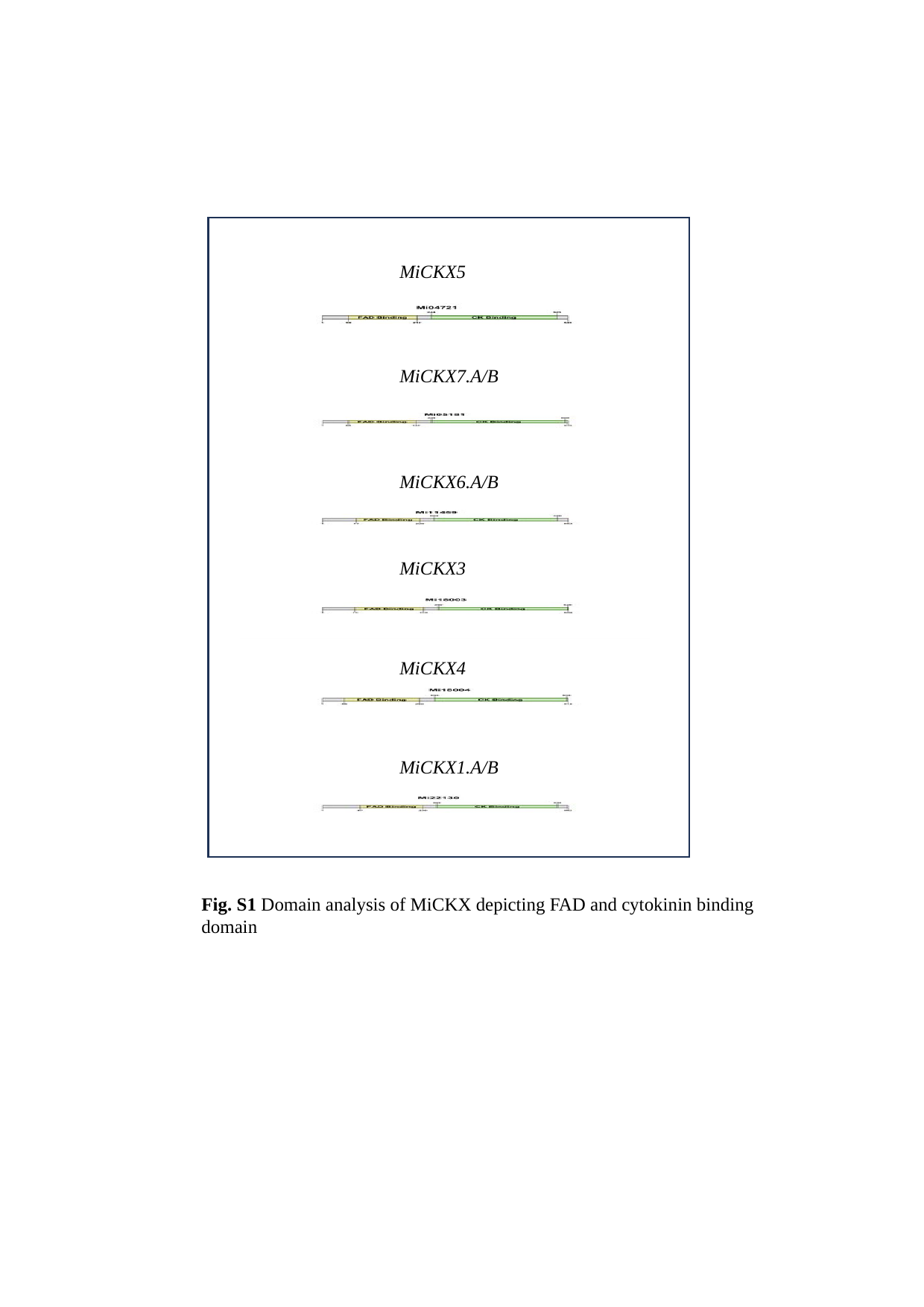

MiCKX5
MiCKX7.A/B
MiCKX6.A/B
MiCKX3
MiCKX4
MiCKX1.A/B
Fig. S1 Domain analysis of MiCKX depicting FAD and cytokinin binding domain

## Slide 2
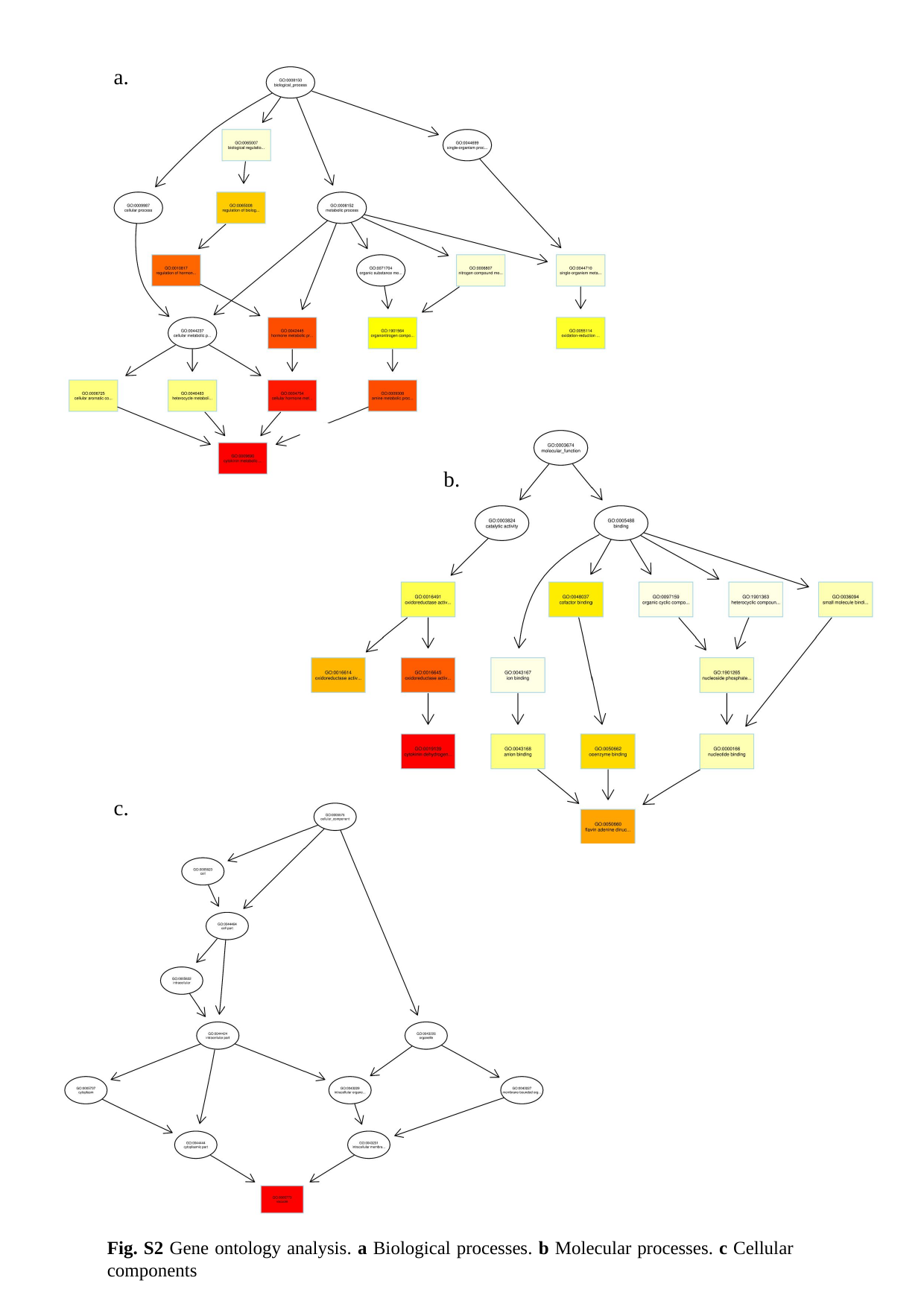

a.
b.
c.
Fig. S2 Gene ontology analysis. a Biological processes. b Molecular processes. c Cellular components

## Slide 3
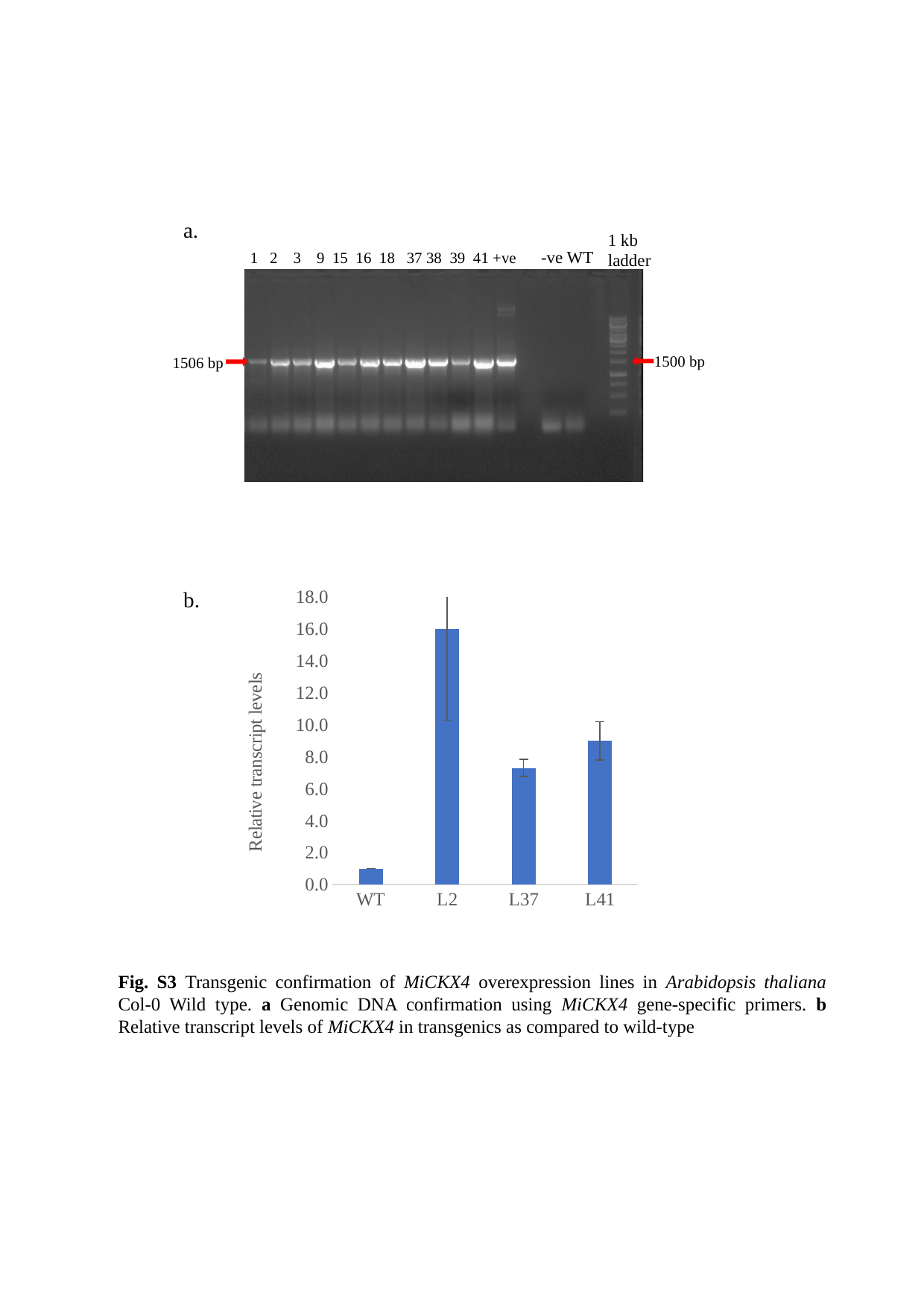

a.
1 kb ladder
-ve WT
1 2 3 9 15 16 18 37 38 39 41 +ve
1500 bp
1506 bp
b.
### Chart
| Category | |
|---|---|
| WT | 1.0 |
| L2 | 16.0 |
| L37 | 7.3 |
| L41 | 9.0 |Fig. S3 Transgenic confirmation of MiCKX4 overexpression lines in Arabidopsis thaliana Col-0 Wild type. a Genomic DNA confirmation using MiCKX4 gene-specific primers. b Relative transcript levels of MiCKX4 in transgenics as compared to wild-type
